# Extrafollicular CD4 T cell-derived IL-10 functions rapidly and transiently to support anti-*Plasmodium* humoral immunity

**DOI:** 10.1371/journal.ppat.1009288

**Published:** 2021-02-02

**Authors:** Fionna A. Surette, Jenna J. Guthmiller, Lei Li, Alexandria J. Sturtz, Rahul Vijay, Rosemary L. Pope, Brandon L. McClellan, Angela D. Pack, Ryan A. Zander, Peng Shao, Linda Yu-Ling Lan, Daniel Fernandez-Ruiz, William R. Heath, Patrick C. Wilson, Noah S. Butler

**Affiliations:** 1 Immunology Graduate Program, University of Iowa, Iowa City, Iowa, United States of America; 2 Department of Medicine, Section of Rheumatology, The Knapp Center for Lupus and Immunology Research, The University of Chicago, Chicago, Illinois, United States of America; 3 Department of Microbiology and Immunology, University of Iowa, Iowa City, Iowa, United States of America; 4 Blood Research Institute, Blood Center of Wisconsin, Milwaukee, Wisconsin, United States of America; 5 Committee on Immunology, The University of Chicago, Chicago, Illinois, United States of America; 6 Department of Microbiology and Immunology, Peter Doherty Institute for Infection and Immunity, University of Melbourne, Melbourne, Australia; 7 ARC Centre of Excellence in Advanced Molecular Imaging, University of Melbourne, Parkville, Australia; University of Oxford, UNITED KINGDOM

## Abstract

Immunity against malaria depends on germinal center (GC)-derived antibody responses that are orchestrated by T follicular helper (TFH) cells. Emerging data show that the regulatory cytokine IL-10 plays an essential role in promoting GC B cell responses during both experimental malaria and virus infections. Here we investigated the cellular source and temporal role of IL-10, and whether IL-10 additionally signals to CD4 T-cells to support anti-*Plasmodium* humoral immunity. Distinct from reports of virus infection, we found that IL-10 was expressed by conventional, Foxp3-negative effector CD4 T cells and functioned in a B cell-intrinsic manner only during the first 96 hours of *Plasmodium* infection to support humoral immunity. The critical functions of IL-10 manifested only before the orchestration of GC responses and were primarily localized outside of B cell follicles. Mechanistically, our studies showed that the rapid and transient provision of IL-10 promoted B cell expression of anti-apoptotic factors, MHC class II, CD83, and cell-cell adhesion proteins that are essential for B cell survival and interaction with CD4 T cells. Together, our data reveal temporal features and mechanisms by which IL-10 critically supports humoral immunity during blood-stage *Plasmodium* infection, information that may be useful for developing new strategies designed to lessen the burden of malaria.

## Author summary

Malaria is associated with the expression of both inflammatory and anti-inflammatory cytokines that together regulate disease severity and the function of parasite-specific immune cells subsets. Emerging data show that the anti-inflammatory cytokine Interleukin (IL)-10 can function to sustain an already established humoral immune response during acute and chronic virus infections. Unexpectedly, our results show that IL-10 functions only within the first 72–96 hours of *Plasmodium* blood-stage infection to promote initial helper T cell and B cell interactions, as well as B cell differentiation and survival. Our studies of the temporal features, spatial relationships, critical cellular sources and mechanisms by which IL-10 promotes humoral immunity during malaria highlight the distinct mechanisms governing anti-malarial immunity. This new information may lead to the identification of novel pathways and approaches for eliciting durable protection against malaria.

## Introduction

*Plasmodium* parasite infections and the disease malaria remain a major burden on global public health with more than 228 million infections and approximately 405,000 deaths occurring annually [1]. While it is well documented that antibodies play an essential role in limiting malarial disease and promoting parasite control [2,3], anti-*Plasmodium* humoral immunity is short-lived, generally leaving individuals living in malaria endemic regions susceptible to re-infection [4–8]. Deficiencies in protective humoral immunity against malaria have been associated with exacerbated pro-inflammatory cytokine responses that include production of type I and type II interferons and TNF [9–13]. The mechanisms by which inflammatory cytokines impair long-lived humoral immunity have been linked to their capacity to limit the development and function of specific immune cell subsets that include T follicular helper (TFH) cells and germinal center (GC) B cells.

TFH critically promote long-lived humoral immunity via secretion of cytokines such as IL-4 and IL-21 and through provision of co-stimulatory signals that aid in the selection and survival of GC B cells [14]. GC B cells, in turn, can differentiate into long-lived plasma cells (LLPC) and memory B cells (MBC) [15]. LLPC-derived antibodies are believed essential for *Plasmodium* parasite clearance during primary infections [16], whereas MBC are postulated to accelerate parasite control upon re-infection [16,17]. The development and function of TFH cells are governed by the transcriptional repressor Bcl6, which promotes the expression of CXCR5, PD-1, and ICOS [18,19], factors that govern homing, localization and co-stimulation of GC B cells. Although the highly pro-inflammatory environment established during malaria may contribute to the inefficient acquisition of long-lived anti-*Plasmodium* immunity, recent data show that anti-inflammatory responses also play a direct and critical role in supporting long-lived pathogen-specific humoral immunity.

IL-10 is a regulatory cytokine and a potent suppressor of inflammation [20,21]. During *Plasmodium* infections, elevated IL-10 has been historically associated with limiting dendritic cell activation, T helper type 1 (TH1) cell activity, and host immunopathology [22,23]. However, we identified that B cell-intrinsic IL-10 signaling is essential for GC B cell differentiation, secretion of protective anti-*Plasmodium* antibodies, parasite control, and host survival (10). More recently, TFH and Foxp3+ T follicular regulatory (TFR)-derived IL-10 was reported to function during established GC responses to support the selection and survival of virus-specific GC B cells [24,25]. TFR cells have additionally been shown to support antigen-specific antibody responses in models of immunization and allergy [26,27]. Thus, IL-10 can either constrain or promote protective immunity, depending on the sources and cellular targets of this regulatory cytokine.

Despite recent data showing the importance of IL-10 in promoting anti-microbial humoral immunity, the critical cellular source, temporal features and capacity for IL-10 to additionally regulate TFH responses to promote protective, anti-*Plasmodium* secreted antibody responses have not been investigated. Moreover, the mechanisms by which IL-10 regulates *Plasmodium*-specific B cell reactions to support humoral immunity have not been addressed. In this report, we explored these key knowledge gaps by genetically and biochemically manipulating IL-10 and IL-10 signaling during a non-lethal *Plasmodium yoelii* murine model of malaria. Distinct from reports of the regulation of anti-viral humoral immunity, our data reveal that during experimental malaria, non-TFH and -TFR CD4 T cells are the critical source of IL-10 that functions prior to the formation of the GC response to promote B cell activation and expression of cell surface proteins necessary for productive interactions with CD4 T cells. Thus, our data highlight how IL-10 may distinctly govern humoral immunity during *Plasmodium* infection and further identify potential opportunities to improve durable immunity against malaria.

## Results

### IL-10 is essential for optimal accumulation and function of *Plasmodium* infection-induced TFH responses

**Fig 1:**
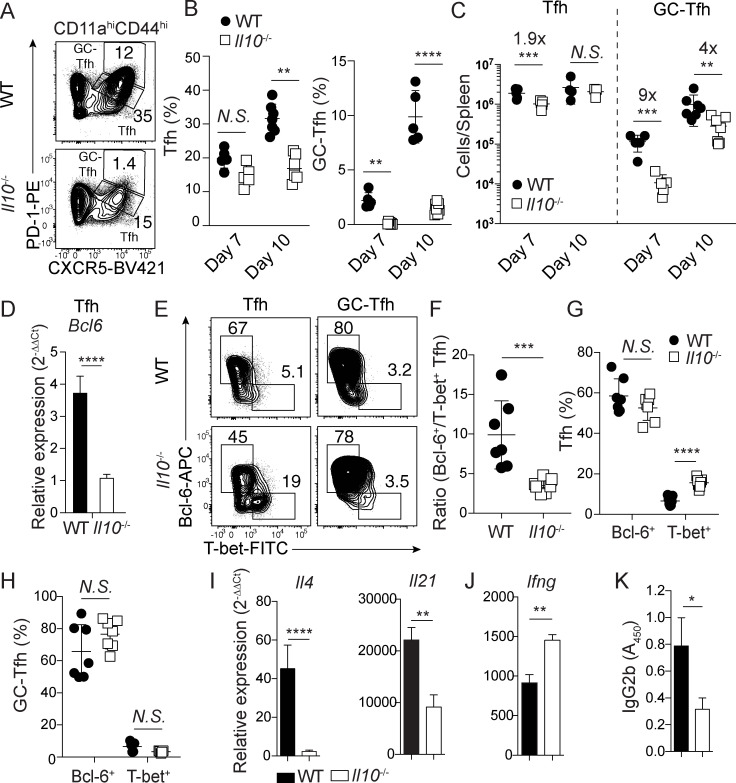
TFH cell responses are numerically and functionally impaired in the absence of IL-10. (**A**-**J**) WT (n = 7) or *il10*^-\-^ (n = 7) C57BL/6 mice were infected with *P*. *yoelii*. Representative plots (**A**) and summary data showing the proportions (**B**) and total numbers (**C**) of TFH (CXCR5^+^PD-1^+^) and GC-TFH (CXCR5^hi^PD-1^hi^) cells on day 7 and 10 p.i. (**D**) TFH cells were sort-purified from WT and *Il10*^-/-^ mice on day 10 p.i. and mRNA levels of *Bcl6* were measured with RT-PCR. Representative plots (**E**) and summary data (**F**) showing the ratio of Bcl-6 expressing versus T-bet expressing TFH cells. (**G,H**) Proportion of Bcl-6 and T-bet expressing in TFH (**G**) and GC-TFH (**H**) cells on day 10 p.i. (**I,J**) GC-TFH cells were sort-purified from WT and *Il10*^-/-^ mice on day 10 p.i. and mRNA levels of *il4*, *il21*, (**I**) and *ifng* (**J**) were determined using RT-PCR. (**K**) GC-TFH cells were co-cultured with naïve B cells in the presence of α-IgM and α-CD3ε and levels of secreted IgG2b antibody were determined by ELISA. Data (means ± S.E.M.) in **A**-**H** are pooled from 2 independent experiments and were analyzed using unpaired, non-parametric Mann-Whitney tests. * *P* ≤ 0.05, ** *P* ≤ 0.01, *** P ≤ 0.001, **** *P* < 0.0001.

B cell-intrinsic IL-10 signaling is critical for promoting GC-dependent control of blood stage *Plasmodium* infection [10]. Whether IL-10 also directly influences GC-TFH differentiation is not known. GC-TFH cells are essential for host survival during experimental malaria [11,16] and their development proceeds via a step-wise process involving upregulation of PD-1, CXCR5, and Bcl6 expression that culminates with the localization of GC-TFH cells in the germinal center [28]. To address the potential effects of IL-10 on TFH development and function during experimental malaria, we first evaluated whether either CXCR5^int^PD-1^int^ TFH or CXCR5^hi^PD-1^hi^ GC-TFH responses were impaired in *Plasmodium*-infected *Il10*^-/-^ mice. On both days 7 and 10 p.i., we observed 4- to 5-fold reductions in the frequencies (**Fig 1A** and **1B**) and 4- to 9-fold reductions in total numbers (**Fig 1C**) of GC-TFH cells in *P*. *yoelii*-infected *Il10*^*-/-*^ mice, compared to *P*. *yoelii*-infected WT mice. Notably, the total numbers of TFH cells in *P*. *yoelii*-infected *Il10*^*-/-*^ mice were reduced less than 2-fold on day 7 p.i. and equivalent on day 10 p.i. (**Fig 1C**), suggesting that IL-10 primarily promotes TFH transition and commitment to the GC-TFH program during blood stage *Plasmodium* infection. Consistent with this, we observed markedly reduced *Bcl6* mRNA expression (**Fig 1D**) and concomitant decreases in the ratios of Bcl6^+^ to T-bet^+^ TFH cells (**Fig 1E–1G**), but not GC-TFH cells (**Fig 1H**), recovered from *P*. *yoelii*-infected *Il10*^-/-^ mice on day 10 p.i. Together, these data support that in the absence of IL-10, *Plasmodium* infection-induced effector TFH cells fail to undergo optimal step-wise differentiation into GC-TFH cells and may instead be skewed towards a T-bet^+^ TH1-like differentiation profile.

Despite observing equivalent Bcl6 and T-bet expression patterns among CXCR5^hi^PD-1^hi^ GC-TFH cells recovered from *P*. *yoelii*-infected WT and *Il10*^*-/-*^ mice, we further tested whether GC-TFH cells that develop in the absence of IL-10 exhibit impaired function through transcriptional profiling and *in vitro* B cell helper assays [29]. Sort-purified CXCR5^hi^PD-1^hi^ GC-TFH cells recovered from *Il10*^-/-^ mice exhibited 20- and 2.5-fold reductions in *il4* and *il21* expression, respectively, compared to GC-TFH cells recovered from *P*. *yoelii*-infected WT mice (**Fig 1I**). Moreover, transcripts encoding IFN-γ, a cytokine that directly impairs anti-*Plasmodium* GC B cell function [10], were upregulated >35% in GC-TFH cells recovered from *Plasmodium*-infected *Il10*^-/-^ mice (**Fig 1J**). As expected, *Il10* mRNA expression was not detected in purified CXCR5^hi^PD-1^hi^ GC-TFH recovered from *Il10*^-/-^ mice (**S1A Fig**). During Tfh:naïve B cell co-culture assays, GC-TFH cells recovered from *Plasmodium*-infected *Il10*^-/-^ mice were 60% less capable of stimulating naïve B cell class-switching and secretion of IgG2b, compared to an equivalent number of cells recovered from WT mice (**Fig 1K**). Collectively, these data support that the step-wise differentiation of GC-TFH cells is impaired in the absence of IL-10 and the reduced number of CXCR5^hi^PD-1^hi^ GC-TFH cells that do develop display characteristics of TH1-like TFH cells (TFH1) observed in *P*. *falciparum*-infected humans [30] and exhibit reduced B cell helper functions. Thus, IL-10 is critical for promoting the optimal accumulation and B cell helper function of GC-TFH cells during experimental malaria.

### Early IL-10R signaling is essential for optimal GC-Tfh accumulation and GC B cell differentiation during experimental malaria

**Fig 2:**
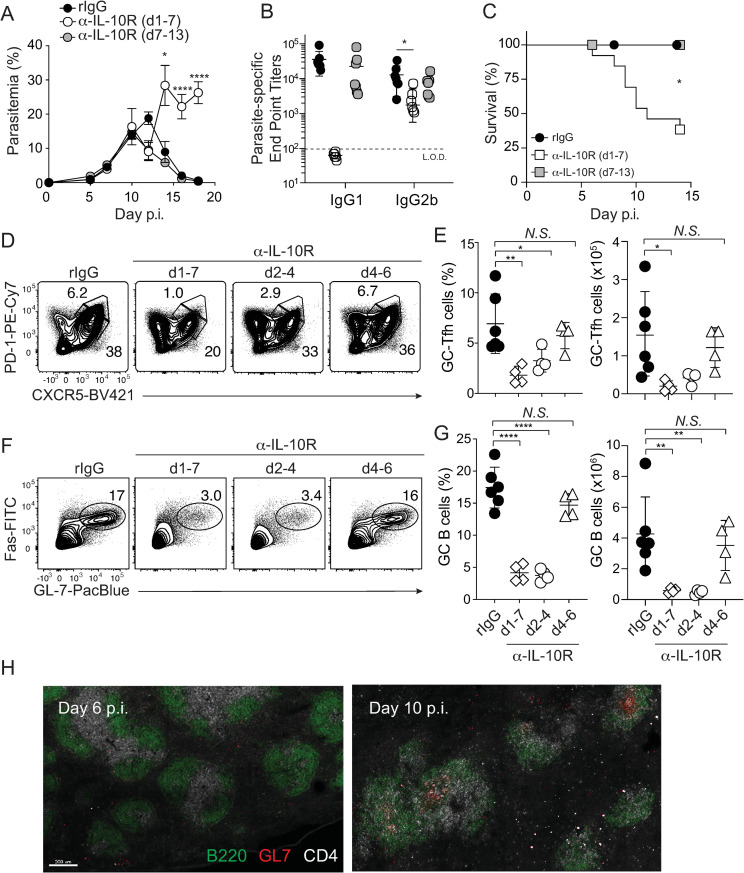
Early IL-10 signaling is critical for humoral immunity. (**A-C**) *P*. *yoelii* infected WT mice were administered α-IL-10R from either days 1–7 p.i. (n = 6), days 7–13 p.i. (n = 7) or rIgG (n = 7). Parasitemia (**A**), parasite lysate-specific end point titers on day 18 p.i. (**B**) and survival (**C**) were evaluated. Data in A-C (means ± S.E.M.) represent n = 3–4 mice/group pooled from 2 independent experiments. *P ≤ 0.05 and ****P ≤ 0.0001 comparing rIgG to α-IL-10R (d1-7). Differences between rIgG and α-IL-10R (d7-13) were not significant in any assay. (**D-G**) Representative plots and summary data showing numbers of GC-TFH cells (**D,E**) and GC B cells (**F,G**) that develop when IL-10R signaling is blocked on the indicated days p.i. (**H**) Groups of WT C57BL/6 mice (n = 3/time point) were infected with *P*. *yoelii* and on day 6 and 10 p.i. spleens were sectioned and stained with α-B220-AF488, clone GL-7-biotin, α-CD4-AF647. The sections were then counter-stained with streptavidin-BV421. Images were processed using IMARIS software. Data in **A** were analyzed by Log-rank (Mantel-Cox) test. Data in **B** were analyzed using unpaired, non-parametric Kruskal-Wallis tests correcting for multiple comparisons using the Dunn’s test. Data in **E** and **G** were analyzed using one-way ANOVA and Dunnett’s multiple comparison tests, with each group compared to rIgG. * *P* ≤ 0.05, ** *P* ≤ 0.01, *** P ≤ 0.001, **** *P* < 0.0001.

Previous studies showed that GC B cell responses and antibody production are absent in mice treated with an anti-IL-10R blocking antibody (clone 1B1.3a) throughout the course of *P*. *yoelii* infection [10], and we observed *Il10* mRNA expression in *Plasmodium*-infection induced GC-Tfh cells (**S1A Fig**). Thus, IL-10 could act during either initial B cell and CD4 T cell activation and differentiation or during the amplification and maintenance phases of anti-*Plasmodium* humoral immune responses. To test whether IL-10 functions during a critical temporal window to support humoral immunity in *P*. *yoelii*-infected mice, we utilized timed delivery of α-IL-10R. Blocking IL-10R signaling from days 7 to 13 p.i. (late) had no impact on either parasite control, parasite-specific secreted antibody responses or host survival (**Fig 2A–2C**). By contrast, IL-10R signaling blockade from days 1 to 7 p.i. resulted in elevated parasite burden (**Fig 2A**), loss of circulating parasite-specific IgG1, 10-fold reductions in circulating parasite-specific IgG2b (**Fig 2B**) and >60% mortality (**Fig 2C**), which mirrors the phenotype of *P*. *yoelii*-infected *Il10*^-/-^ mice (**S1B–S1F Fig**). Strikingly, blocking IL-10R signaling between days 2 and 4 p.i., but not after day 4 p.i., was sufficient to abrogate GC-TFH (**Fig 2D** and **2E**) and GC B cell (**Fig 2F** and **2G**) responses by day 9 p.i. Confocal imaging further revealed that fully formed GC structures are not yet detectable within the first 6 days p.i. (**Fig 2H**), suggesting that the temporal window in which IL-10 is critical for sustaining humoral immunity precedes the organization of GC structures. Collectively, these data support that IL-10R signaling functions within the first 2 to 4 days of blood-stage *Plasmodium* infection to promote humoral immunity, likely during the activation phase when CD4^+^ helper T cells and B cells make initial encounters prior to their entry into B cells follicles and their orchestration of GC responses.

### Extrafollicular Foxp3-negative CD4 T cells are a critical source of IL-10 supporting anti-*Plasmodium* humoral immunity during the first 4 days of *P*. *yoelii* infection

**Fig 3:**
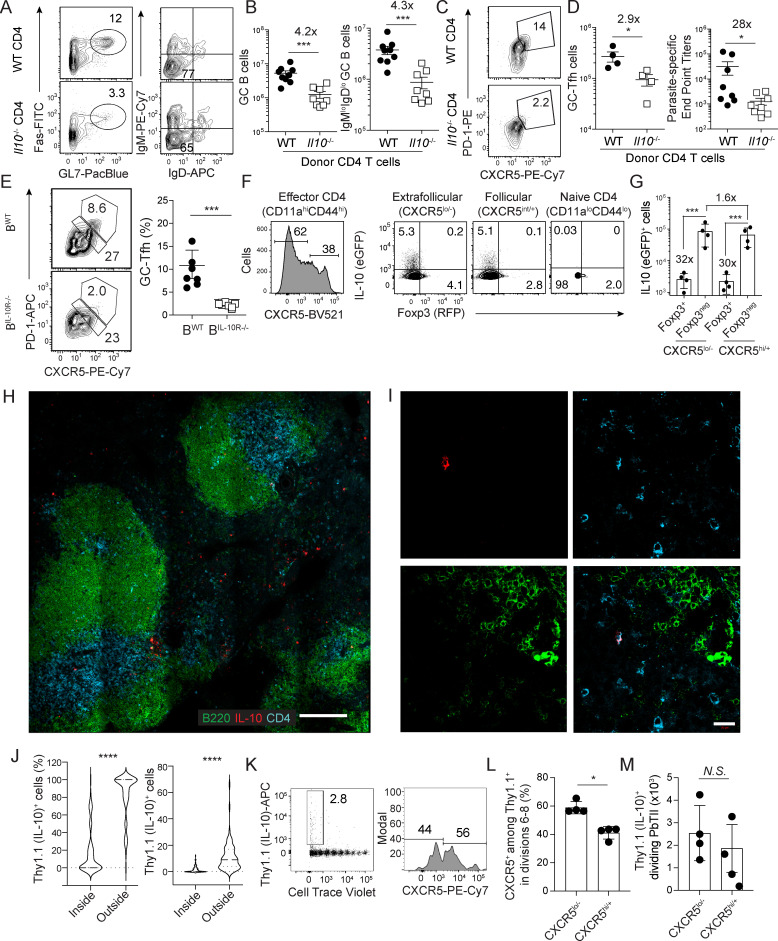
Extrafollicular, conventional effector CD4 T cells are the primary source of IL-10 during the first 4 days of *P*. *yoelii* infection. (**A**-**D**) 2x10^6^ WT or *Il10*^-/-^ CD4 T cells were adoptively transferred into *Tcra*^-/-^ mice 1 day prior to infection with 10^6^ *P*. *yoelii* pRBC. Spleens were harvested on day 18. Representative plots (**A**) and summary data (**B**) of GC B cells and class-switched GC B cells. Representative plots (**C**) and summary data of GC-TFH cells (**D**, left) and parasite-lysate specific IgG2b end point titers (**D**, right) on day 18 p.i. Data (means ± S.E.M.) in B,D are from n = 9 WT and n = 8 *Il10*^-/-^ recipients pooled from 2 independent experiments and analyzed using unpaired, non-parametric Mann-Whitney tests. (**E**) Competitive mixed bone marrow chimeric mice were generated in which B cells were either WT (B^WT^, n = 7) or specifically lacked *Il10rβ* (B^IL-10R^, n = 10). Representative plots (left) and summary data (right) of TFH cells and GC-TFH cells on day 21 p.i. Data (means ± S.E.M.) were analyzed using unpaired, non-parametric Mann-Whitney tests and are representative of 2 independent experiments. (**F**) Representative plots showing IL-10 expression by CXCR5^+^ and CXCR5^neg^ conventional (Foxp3^-^) and regulatory (Foxp3^+^) effector CD4 T cells on day 4 p.i. (**G**) Total numbers of each subset in the spleen on day 4 p.i. Data in F,G (means ± S.D.) and were analyzed using unpaired, non-parametric Mann-Whitney tests and are representative of 2 independent experiments (n = 4 mice). (**H-I**) Histological examination of IL-10 production on day 4 p.i. in the spleens of 10BiT mice stained with anti-B220-AF488 (green) anti-CD4-AF647 (blue) and anti-Thy1.1-PE/Dazzle (red). Data in H-I are representative of 4 independent tiled scans acquired from n = 4 10Bit spleens. (**J**) Summary of the proportion (left) and absolute number (right) of Thy1.1 (IL-10)^+^ cells that localized either outside or within B cell follicles quantified from 25 independent scans and analyzed using unpaired, non-parametric Mann-Whitney tests. (**K-M**) 1 x 10^6^ 10BiT PbTII cells were CTV-labeled and adoptively transferred into C57BL/6 mice 1 day prior to infection with *P*. *yoelii* pRBC. Spleens were harvested on day 4. Representative plots (**K**), and summary data of frequency (**L**) and total numbers (**M**) of CXCR5^+^ and CXCR5^neg^ IL-10-expressing PbTII cells. Data (means ± S.D.) in L,M are representative of 2 independent experiments (n = 4) and were analyzed using unpaired, non-parametric Mann-Whitney tests. Scale bars in H and I represent 200 (H) and 20 μm (I), respectively. * *P* ≤ 0.05 *** P ≤ 0.001 **** P ≤ 0.0001.

Multiple cells types, including B cells, express IL-10 during experimental *Plasmodium* infection (reviewed in [31,32]), yet CD4 T cells are reportedly the source of IL-10 that limits severe inflammation and promotes host survival [22,33]. We observed (**S2A**–**S2C Fig**) that CD4 T cells represent the majority (~60–75%) of IL-10-expressing cells in *P*. *yoelii*-infected 10Bit mice on day 4 p.i., which report surface Thy1.1 as a surrogate for IL-10 expression [34]. Smaller proportions (~10%) of CD3+CD4- cells (CD8 T cells) express IL-10, with substantial variability (10–50%) among the remainder of the cells. To formally test whether CD4 T cells serve as an essential source of IL-10 that sustains anti-*Plasmodium* humoral immunity, we adoptively transferred naïve CD4 T cells from either WT or *Il10*^-/-^ donor mice into *Tcra*^-/-^ mice and infected recipients with *P*. *yoelii* one day later. Engraftment of WT and *Il10*^-/-^ donor-derived CD4 T cells in recipient mice was equivalent (**S2D Fig**). Compared to recipients of WT CD4 T cells, recipients of *Il10*^-/-^ CD4 T cells exhibited 4-fold reductions in the number of class-switched (IgD^lo^IgM^lo^) GC B cells (**Fig 3A** and **3B**) and 3-fold reductions in the number of GC-TFH cells (**Fig 3C**). In line with these cellular changes, we also observed nearly 30-fold reductions in parasite-specific IgG2b titers in recipients of *Il10*^*-/-*^ CD4 T cells (**Fig 3D**). Thus, despite multiple potential cellular sources of IL-10, GC B cell, GC-TFH cell and parasite-specific antibody impairments observed in *Plasmodium*-infected mice treated with α-IL-10R antibody were phenocopied by infection of mice harboring *Il10*^*-/-*^ CD4 T cells.

Given the profound losses in GC-TFH cell number and function in *Plasmodium*-infected *Il10*^-/-^ mice, and the known inhibitory effects of CD8 T cell-intrinsic IL-10 signaling during experimental *Plasmodium* infection [35], it was possible that CD4 T cell-intrinsic IL-10 signaling additionally supports step-wise TFH induction or acquisition of the GC-TFH program during experimental malaria. To directly evaluate TFH differentiation among IL-10-responsive and non-responsive CD4 T cells, we established an experimental system where WT and *Il10rβ*-/- cells are subject to equivalent inflammatory and antigenic stimuli and accessory cell functions. To do this, we generated and infected WT:*Il10rβ*^-/-^ (1:1) mixed bone marrow chimeric mice to study TFH and GC-TFH development among both WT and *Il10rβ*^-/-^ CD4 T cells in the same *P*. *yoelii*-infected host. By day 10 p.i., we observed that the frequencies of both TFH and GC-TFH cells were equivalent among *Il10rβ*^-/-^ and WT CD4 T cells in the same host (**S2E Fig**). Thus, the abrogation of CD4 T cell-intrinsic IL-10 signaling is not sufficient to impede the *in vivo* development of either GC-TFH cells, GC B cell reactions, or anti-*Plasmodium* humoral immunity.

TFH cell commitment to GC-TFH differentiation depends on the concomitant differentiation and function of GC B cells [14,19]. Thus, we next hypothesized that impaired acquisition of the GC-TFH program in *Plasmodium*-infected *Il10*^-/-^ mice (**Fig 1**) may reflect the lack of GC B cell responses in mice with blunted IL-10 signaling [10]. To formally test this, we evaluated TFH and GC-TFH development in *P*. *yoelii*-infected *μMT*^-/-^:*Il10rβ*^-/-^ (8:2) competitive mixed bone marrow chimeric mice in which *Il10rβ*^-/-^ deficiency is restricted to the B cell compartment (B^IL-10R-/-^). Notably, *Plasmodium*-infected B^IL-10R-/-^ mice harbor ~10-fold fewer GC B cells compared to infected B^WT^ mice (10). In support of our hypothesis, the proportions of GC-TFH cells were reduced by 4 to 5-fold in *P*. *yoelii*-infected B^IL-10R-/-^ mice (**Fig 3E**). Together, these data support a model in which CD4^+^ T cell derived IL-10 acts directly on B cells to support GC B cell differentiation or accumulation, which in turn reinforces GC-TFH development and function.

Both Foxp3^+^ T follicular regulatory (TFR) and conventional (Foxp3-negative) TFH cells serve as critical sources of IL-10 that sustain antiviral GC responses [24,25]. To determine which CD4 T cell subsets express IL-10 during *Plasmodium* infection, we analyzed CD4 T cells recovered from *P*. *yoelii*-infected IL-10-eGFP/Foxp3-RFP double-reporter mice within the 96-hour window that we established as critical by timed delivery of α-IL-10R (**Fig 2**). The frequency and number of PD-1-expressing CD4 helper T cells were expectedly low at this early time point, so we focused on evaluating Foxp3^+^ and Foxp3^neg^ activated (CD11a^hi^CD44^hi^) CD4 T cells exhibiting phenotypic characteristics of either extrafollicular (CXCR5^lo/-^) or follicular (CXCR5^int/+^) localization. Among both CXCR5^lo/-^ and CXCR5^int/+^ effectors, we found that ~5% of both conventional (Foxp3^neg^) and regulatory (Foxp3^+^) CD4 T cells expressed IL-10/eGFP (**Fig 3F**). However, the total number of conventional IL-10/eGFP^+^ cells exceeded regulatory IL-10/eGFP^+^ cell subsets by ~30-fold (**Fig 3G**). Confocal imaging of the spleens of *P*. *yoelii*-infected Foxp3-RFP/IL-10eGFP double reporter mice further supported that IL-10 signal did not appreciably overlap with Foxp3 (**S3A Fig**). Histological analyses of the spleens of *P*. *yoelii*-infected 10Bit reporter mice also showed that IL-10-expressing cells primarily localized to CD4 T cells outside the border of B cell follicles on day 4 p.i. (**Fig 3H–3J**, and **S3B** and **S3C Fig**), results that were consistent with direct histological staining for IL-10 in *P*. *yoelii*-infected WT mice (**S3D** and **S3E Fig**).

The rapid and essential provision of IL-10 by conventional CD4 T cells raised the possibility that *Plasmodium*-specific effector CD4 T cells may be rapidly programed to express IL-10 within days of an initial *Plasmodium* blood-stage infection. To test this, we crossed rodent *Plasmodium*-specific CD4 T cell receptor transgenic mice (PbTII, [36]) with the 10BiT reporter mice. An appreciable fraction (~3%) of parasite-specific 10BiT PbTII cells expressed Thy1.1 within 4 days of their initial activation, which was further linked to multiple rounds of cell division (**Fig 3K**), suggesting IL-10 expression was programmed during their proliferative expansion. Similar to the endogenous response, IL-10 expressing PbTII cells were both CXCR5^lo/-^ and CXCR5^int/+^ (**Fig 3L** and **3M**). Together, these data show that at 96 hours p.i., the majority of IL-10-expressing cells exhibit characteristics of conventional, Foxp3^neg^ effector CD4 T cells and localize outside B cell follicles, suggesting that fully differentiated GC-TFH and -TFR subsets are unlikely to serve as critical sources of early IL-10 that potentiate anti-*Plasmodium* humoral immunity. These data also highlight the rapidity with which *Plasmodium*-specific effector CD4 T cells can be programmed to express IL-10 during malaria.

### IL-10 is necessary for optimal B cell expression of molecules that support CD4 T cell-B cell interactions

**Fig 4:**
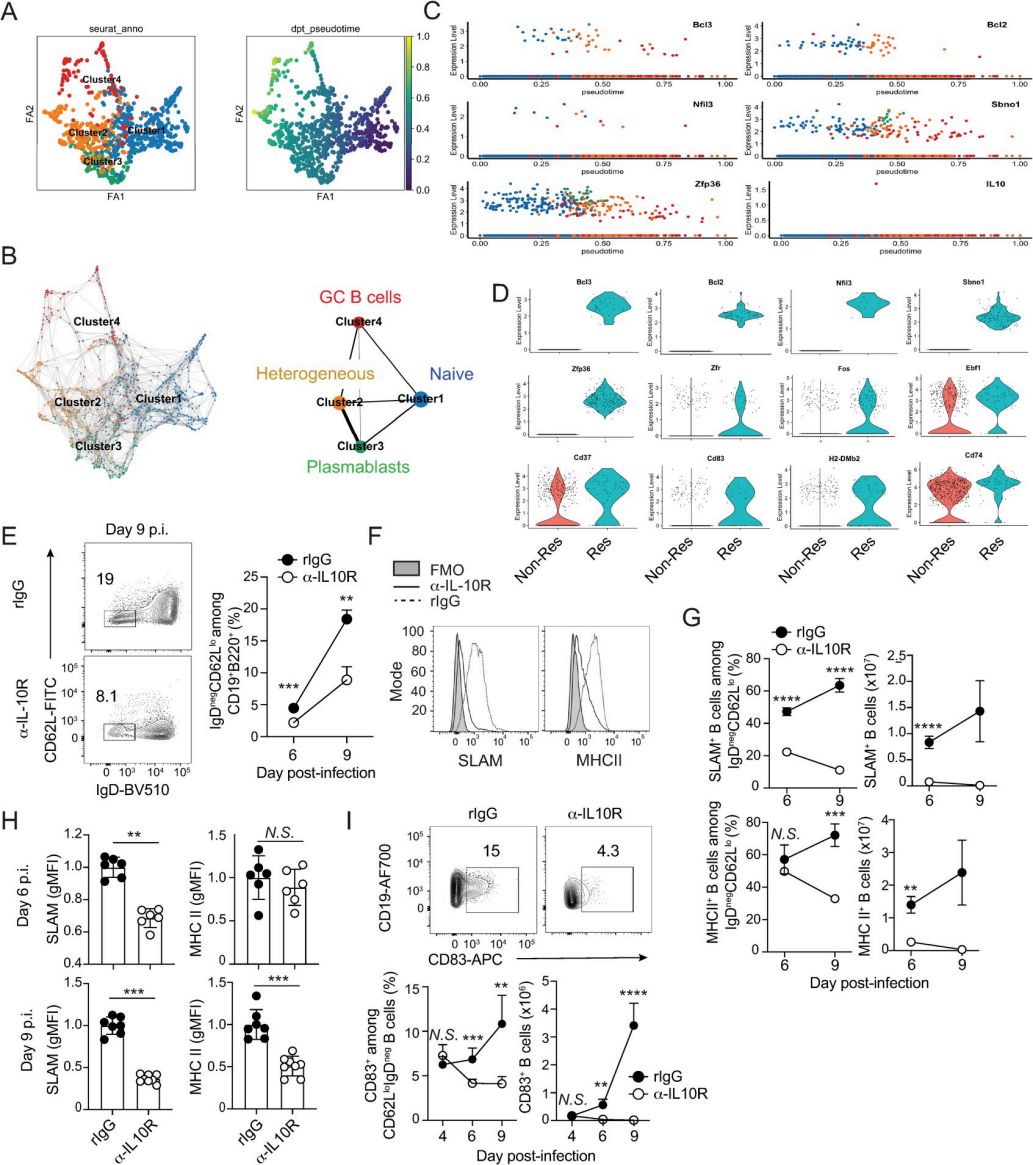
IL-10 promotes optimal expression of SLAM, CD83 and MHC II by B cells during experimental malaria. (**A-D**) Ten days after infection with 10^6^ *P*. *yoelii* pRBC, splenic B cells were sort purified from WT mice (n = 3) and subjected to single cell RNA-sequencing. (**A**) Single cells visualized on a 2D ForceAtlas2 (FA) embedding. Four cell clusters were represented by different colors to the left panel and pseudotime of cells were indicated by gradient colors to the right panel. (**B**) PAGA-initialized single-cell embeddings (relationship network among individual cells, left panel) and PAGA graphs (relationship network among cell clusters, right panel) of all B cells and clusters. (**C**) Transcriptome expression levels of 6 key marker genes (*Bcl3*, *Bcl2*, *Nfil3s*, *Sbno1*, *Zfp36*, *Il10*) of IL-10/STAT3 pathway along the pseudotime. Coloration of blue, orange, green and red corresponds to the four clusters shown in panel B. (**D**) Transcriptome expression levels of 12 marker genes: *Bcl3*, *Bcl2*, *Nfil3*, *Sbno1*, *Zfp36*, *Zfr*, *Fosb*, *Ebf1*, *Cd37*, *Cd83*, *H2-dmb2 and Cd74* among early IL-10-responsive (Res) and early IL-10 non-responsive (Non-Res) B cells. (**E-I**) *P*. *yoelii* infected WT mice were administered either rIgG (n = 6) or α-IL-10R (n = 6–8) from days 2–4 p.i. (**E**) Representative plots and summary data show the frequency of activated CD19^+^B220^+^IgD^neg^CD62L^lo^ B cells. (**F**) Representative histograms showing expression of SLAM and MHC II on activated B cells recovered from rIgG- (dashed line) and α-IL-10R-treated (solid line) mice on days 6 p.i. (**G**) Summary data showing frequency and total numbers of SLAM^+^ (top panels) and MHCII^+^ (bottom panels) activated B cells on days 6 and 9 p.i. (**H**) Summary data showing geometric mean fluorescence intensity (gMFI) of SLAM and MHC II staining on days 6 and 9 p.i. (**I**) Representative plots from day 9 p.i. and summary data show the frequency, total number and gMFI of CD83 on activated B cells. gMFI data are normalized to the average of control group gMFI. Data (means ± S.E.M.) in **E** were analyzed using unpaired, non-parametric Mann-Whitney tests. Data (means ± S.D.) Data in **G**, **H** and **I** were analyzed using unpaired, non-parametric Mann-Whitney test and are representative of 4 independent experiments. ** *P* ≤ 0.01, *** P ≤ 0.001, **** *P* < 0.0001.

Recent reports showed that B cells can serve as an important APC for priming and promoting TFH responses during both viral [37] and blood-stage *Plasmodium* [38] infections. Thus, we next evaluated whether early (days 2–4 p.i.) provision of IL-10 modulates either the transcriptional profile or APC phenotype of B cells, as this may in turn affect their ability to initiate and reinforce a TFH cell program. To gain initial insight, we performed single cell RNA sequencing on B cells recovered from *P*. *yoelii*-infected mice on day 10 p.i. and constructed a pseudotime projection that revealed four distinct clusters of B cells representing bystander/naïve B cells (Cluster 1), transcriptionally heterogeneous B cells (Cluster 2), plasmablasts (Cluster 3) and GC B cells (Cluster 4) (**Fig 4A** and **4B**). We mapped onto this projection the expression of five genes (*Bcl2*, *Bcl3*, *Nfil3s*, *Sbno1* and *Zfp36*) that are reported to be IL-10/STAT3-responsive (reviewed in [39]) and identified that each was induced relatively early (0 < 0.5) in the pseudotime projection (**Fig 4C**). We then binned cells as either early IL-10 responders (Res) or early non-IL-10 responders (Non-Res) and examined the top twenty differentially expressed genes (DEG) among these two B cell sub-populations, both of which derive primarily from clusters 1 and 2. In addition to the five known IL-10/STAT3-responsive genes, several transcriptional regulators (*Zfr*, *Fos*, *Ebf1*) and signaling factors important for humoral immunity (*Cd37*) [40], as well as genes encoding CD83 (*Cd83*) and MHCII beta chain (*H2dmb2*) and Ag-processing machinery (*Cd74*), were more highly expressed among early IL-10 responders, compared to early non-responders (**Fig 4D**).

The differential expression of anti-apoptotic and pro-survival genes *Bcl2* and *Bcl3* are consistent with a proliferation and or survival advantage among early IL-10 responder B cells. In line with this, we recovered 40% fewer Ki67^+^ IgD^neg^CD62L^lo^ activated B cells from mice treated with anti-IL-10R from days 2–4 p.i. (**S4A** and **S4B Fig**) and these cells exhibited reduced Ki67 gMFI (**S4C Fig**). Reduced marks of B cell proliferation in anti-IL-10R-treated mice further translated to reduced numbers of activated (CD19^+^B220^+^IgD^neg^CD62L^lo^) B cells by day 6 p.i., with these differences further magnified by day 9 p.i. (**Fig 4E**). Notably, the differential expression of *Cd83*, *H2dmb2* and *Cd74* raised the possibility that early IL-10 signaling in B cells responding to *Plasmodium* infection also promote the expression of genes critical for productive, sustained interactions with CD4 helper T cells. IL-10 is known to promote MHCII expression via the downregulation of MARCH1 in B cells [41] and the expression of MHCII and the cell-cell adhesion protein signaling lymphocytic activation molecule (SLAM) [42] have been shown to be regulated by the activity of CD83 [43,44] and IL-10 signaling [45], respectively. Although, we found no evidence that *March1* mRNA was differentially expressed among follicular B cells in the presence or absence of IL-10 (**S4D Fig**), blocking IL-10 signaling from days 2–4 p.i. revealed 3- to 6-fold reductions in the frequencies of MHCII and SLAM-expressing B cell among the activated (CD19^+^B220^+^IgD^neg^CD62L^lo^) B cell populations (**Fig 4F** and **4G**). The per cell expression of these molecules was also reduced by 50% in the absence of early IL-10 signaling (**Fig 4H**). Moreover, whereas the frequencies and total numbers of CD83^+^ B cells were similar on day 4 p.i., CD83^+^ activated B cells were nearly undetectable by day 9 p.i. in mice treated with α-IL-10R mAb (**Fig 4I**), which was in agreement with the MHCII expression data. In addition to our antibody blockade approaches, we also found that *P*. *yoelii*-infected *Il10*^-/-^ mice recapitulated these B cell phenotypes (**S4E** and **S4F Fig**), supporting that CD83 may act, in part with IL-10, to regulate MHCII and SLAM expression on B cells. Collectively, our data support a model in which conventional, extrafollicular CD4 T cell-derived IL-10 functions within the first four days of *Plasmodium* infection to directly promote B cell activation, proliferation and expression of molecules necessary for their survival and capacity to reinforce the step-wise differentiation of TFH cells and subsequent generation of productive GC responses and anti-malarial humoral immunity.

## Discussion

IL-10 is a pleiotropic cytokine that exhibits a variety of roles in regulating immunity and is regarded as critical for limiting malaria-associated immunopathology, severe disease, and mortality [23,46]. Recently, we and others have defined new *in vivo* roles for IL-10 by showing that IL-10 can act directly on B cells to support anti-microbial GC B cell responses, humoral immunity, and pathogen clearance [10,24,25], underscoring the context-dependent mechanism by which IL-10 regulates anti-microbial immunity. In the current study, we sought to identify if a critical temporal window exists for IL-10, the cell subset(s) responsible for provision of IL-10 to B cells, and the mechanism by which IL-10 functions to promote B cell responses and humoral immunity during experimental malaria.

Here we provided data supporting that conventional (Foxp3^neg^), extrafollicular CD4 T cell-derived IL-10 acts during the first four days of *Plasmodium* blood stage infection to promote survival and stimulate B cell expression of MHC II, SLAM and CD83, molecules that sustain TFH and GC B cell responses and potentiate protective humoral immunity. Consistent with our data, recent work has demonstrated SLAM-mediated stable interactions between TFH and GC B cells are critical for the development of LLPC populations [42]. Our experiments also show that TFH responses develop independently of CD4 T cell-intrinsic IL-10 signaling, supporting that the lack of TFH responses in mice deficient in IL-10 or IL-10 signaling is primarily due to lesions in early B cell activation events that are critical for the coordinate differentiation of both TFH and GC B cell populations.

Our finding that IL-10 is critical during the first 96 hours of *Plasmodium* blood-stage infection is consistent with the notion that B cells must respond to IL-10 well before anatomical formation of germinal centers within B cell follicles, which are only histologically detectable in our system by day ~10 p.i. Our results stand in striking contrast to studies of acute and chronic virus infections. During an acute virus infection, TFR-derived IL-10 is important for regulating Foxo1 activity and the capacity of GC B cells to down-regulate CXCR4 and cycle between the DZ and LZ of the GC [24]. During chronic virus infection, mature populations of TFH cells served as a critical source of IL-10 to promote humoral immunity [25], although the specific mechanisms by which IL-10 programs either B cell fate or function were not formally explored in that report. Thus, during either acute or chronic virus infection, it appears that IL-10 primarily functions to sustain an already established GC response. By comparison, our α-IL-10R blocking studies fail to support a role for IL-10 after day 6 p.i. Thus, rather than provision of IL-10 by TFH or TFR cells and modulation of mature GC B cell biology, we show that parasite-specific, conventional effector CD4 T cell-derived IL-10 plays an essential role in shaping the earliest stages of B cell activation and promoting humoral immunity.

We previously reported that early type I IFN signaling (days 0 to 4 p.i.) promoted the development of IL-10^+^IFN-γ^+^ T regulatory 1 (Tr1) populations that during the second week of infection functioned to constrain anti-*Plasmodium* humoral immunity [13]. The stimuli that program early IL-10 expression in Plasmodium-specific CD4 T cells, the formal relationship of these cells to Tr1 cells and their potential induction by type I IFN signaling remain to be investigated. Nevertheless, our new results highlight that IL-10 exhibits temporally evolving roles that can either promote or constrain humoral immunity depending on the cellular source(s), timing and the context of other cytokines, including IFN-γ. Our experiments also do not rule out potentially critical functions for IL-10 during secondary humoral immune reactions mediated by MBC. IL-10-secreting CD4 T cells are disproportionately lost from the memory CD4 T cell pool following resolution of experimental blood stage *Plasmodium* infection [47]. Moreover, IL-10-nonproducing memory CD4 T cells rapidly upregulated IL-10 expression during recall, with reported potent inhibitory effects on CD4 T cell effector functions during secondary infection [47]. Thus, determining whether IL-10 exerts potentially distinct and non-overlapping roles during memory TFH and MBC reactivation will be of interest.

Functional interrogation of TFH cells in our experiments also revealed new and unexpected results that are likely linked to our observed defects in B cell activation. First, while our studies largely excluded a CD4 T cell-intrinsic role for IL-10 signaling, TFH cells recovered from *Plasmodium*-infected *Il10*^*-/-*^ mice were impaired in their capacity to express transcripts for IL-21 and IL-4. While activated B cells are able to migrate to the interfollicular zone independent of IL-4 and IL-21 signaling, their movement from the interfollicular zone into the GC is IL-21 dependent [48], and our data showing the reduced capacity of TFH to produce IL-21 may additionally explain why GC are not efficiently formed and follicular organization is not well maintained. Second, we observed markedly elevated IFN-γ mRNA expression in TFH recovered from *Il10*^-/-^ mice. TFH cells are known to express cytokines associated with other T helper subsets, including the TH1-associated cytokine IFN-γ [19,49]. Notably, TH1-like TFH cells appear to be preferentially expanded during human *Plasmodium* infections and are reported to provide inferior B cell help, relative to conventional TFH cells [30]. These concepts further dovetail with our recent report showing that IFN-γ can function via B cell-intrinsic mechanisms to impair GC B cell formation and function [10]. Thus, it is likely that IL-10 also promotes humoral immune responses by suppressing the development of TFH1 responses and IFN-γ production. Studies designed to examine temporally evolving roles of IFN-γ during clinical and experimental malaria are warranted.

In conclusion, our data support a model in which conventional CD4 T cell-derived IL-10 signaling acts within the interfollicular zone during the early priming phase of the anti-*Plasmodium* immune response to promote expression of molecules (e.g. MHCII, SLAM, CD83) that are critical for B cell survival and initial CD4 T cell and B cell conjugate formation and the step-wise differentiation and developmental commitment of TFH cells. These data may also inform our understanding of how IL-10 may regulate GC-derived humoral immunity during other infections characterized by highly inflammatory environments that are counterbalanced by IL-10, and such information may lead to the identification of novel pathways and approaches for eliciting durable, protective humoral immunity.

## Materials and methods

### Ethics statement

All animal studies were done in accordance with the principles set forth by the Animal Welfare Act and the National Institutes of Health guidelines for the care and use of animals in biomedical research. All animal studies were reviewed and approved by the University of Iowa Institutional Animal Care and Use Committee under the Animal Welfare Assurance permit D16-00009/A3021-01 and protocol 6121924.

### Mice, infections, and biologics

C57BL/6J WT, *Il10*^-/-^, *Il10rβ*^/-^, μMT, *IL10*-eGFP (Vert-X) [50], Foxp3-RFP (FIR) [51], and *Tcra*^-/-^ [52] mice (6-to-8 weeks old) were purchased from Jackson Laboratories. PbTII mice have been described [36]. 10BiT reporter mice were provided by Dr. Casey Weaver (UAB). The University of Iowa IACUC approved all experiments. *Plasmodium yoelii* clone 17XNL was obtained from MR4 (ATCC). Infections were initiated by a serial transfer of 10^6^ *P*. *yoelii* parasite-infected red blood cells (pRBCs) i.v. Parasitemia was measured by detecting RNA and DNA content in Ter119^+^ red blood cells via flow cytometry every 2–3 days starting on day 5 post-infection (p.i.), as previously described [53]. For IL-10R blockade, WT mice were injected i.p. with 200 μg of anti-IL-10R (1B1.3a, Bioxcell) or rIgG on the indicated days.

### Flow cytometry

Mouse splenocytes were subjected to red blood cell lysis and stained with fluorescently labeled antibodies. Antibodies used for flow cytometric analyses included anti-mouse CD19 (clone 1D3 or 6D5), anti-mouse T and B cell activation marker (clone GL7), anti-mouse Fas (clone Jo2), anti-mouse IgM (clone RMM-1), anti-mouse IgD (clone 11-26c.2a), anti-mouse SLAM (clone TC15-12F12.2), anti-mouse CD83 (clone Michel-19), anti-mouse MHC II (clone M5/114.15.2), anti-mouse CD86 (clone GL-1), anti-mouse CD62L (clone MEL-14), anti-mouse CD23 (clone B3B4), anti-mouse CD21/35 (clone 7E9) anti-mouse CD4 (clone GK1.5), anti-mouse CD11a (clone M17/4), anti-mouse CD44 (clone IM7), anti-mouse CXCR5 (clone 2G8), anti-mouse PD-1 (clone RMP1-30), anti-mouse CD45.1 (clone A20), anti-mouse CD45.2 (clone 104), anti-mouse T-bet (clone 4B10), anti-mouse Foxp3 (clone MF-14) and anti-mouse Bcl-6 (clone K112-91). All antibodies were obtained from Tonbo, eBioscience, BD Biosciences, and Biolegend. Transcription factor staining was done using Foxp3/transcription factor staining buffer (Tonbo Biosciences). Data were acquired with either a Stratedigm S1200Ex, BD LSR Fortessa, or BD LSR II flow cytometer and analyzed using FlowJo software (TreeStar, Inc., Ashland OR).

### ELISA

Plates (Nunc) were coated with 18 μg/ml *P*. *yoelii* infected red blood cell lysate and blocked with 2.5% BSA + 5% normal goat serum. Serum samples were serially diluted and parasite lysate-specific antibodies were detected by HRP-conjugated goat anti-mouse IgM, IgG, IgG1, and IgG2b. The SureBlue reserve TMB Kit (KBL) was used as a substrate and absorbance was assessed with a Spectra Max 340 (Molecular Devices). End point titers were extrapolated from sigmoidal 4PL (where X is log concentration) standard curve for each sample. Limit of detection (LOD) is defined as the mean plus 2 S.D. of the O.D. (A_450_) signal recorded using naïve mouse sera. All calculations were performed in Prism 6 (GraphPad).

### RNA preparation and Real-time PCR

Total RNA was isolated using TriReagent (Sigma-Aldrich). cDNA was synthesized with SuperScript III First Strand Synthesis Kit (Invitrogen) and amplified using Fast Start Universal SYBR Green Master Rox (Roche) and an Applied Biosystems 7500 FAST real-time PCR machine (Life Technologies) Relative gene expression, normalized to naïve CD4 T cells, was calculated utilizing the 2^-ΔΔCt^ method. *Il10* and *March1* were expressed as a ratio of target to *Hprt* Ct. Primer sequences: *Bcl6* 5’-ACTGGAGAGAAGCGTACCC-3’ and 5’-AAGTCGCAGTTGGCTTTTGT-3’; *Il4* 5’-GGCATTTTGAACGAGGTCAC-3’ and 5’-AAATATGCGAAGCACCTTGG-3’; *Il10* 5’- CGGGAAGACAATAACTGCACCC-3’ and 5’- CGGTTAGCAGTATGTTGTCCAGC-3’; *Il21* 5’-CGCCTCCTGATTAGACTTCG-3’ and 5’-TGGGTGTCCTTTTCTCATACG-3’; *Ifng* 5’-CACGGCACAGTCATTGAAAG-3’ and 5’-GTCACCATCCTTTTGCCAGT-3’; *Hprt* 5’-TCCTCCTCAGACCGCTTTT-3’ and 5’-CCTGGTTCATCATCGCTAATC-3’; *March1* 5’- AAGAGAGCCCACTCATCACACC-3’ and 5’-ATCTGGAGCTTTTCCCACTTCC-3’.

### T cell:B cell co-cultures

To evaluate GC-Tfh function, 50,000 GC-Tfh cells (CD4^+^CD11a^hi^CD44^hi^CXCR5^hi^PD-1^hi^) were sort purified from WT and *Il10*^-/-^ *P*. *yoelii* infected mice on day 10 p.i. and co-cultured with 50,000 naïve (CD19^+^IgD^hi^) B cells for 7 days in the presence of 5 μg/ml of anti-CD3ε (eBioscience) and 5 μg/ml of goat anti-mouse IgM (Jackson Immunoresearch). Supernatants were harvested on day 7, diluted 1:4, and incubated overnight in ELISA plates coated with anti-IgG. Plates were blocked and developed as described above.

### CD4 T cell adoptive transfer

2x10^6^ CD4 T cells from either WT or *Il10*^-/-^ mice were adoptively transferred to *Tcra*^*-/-*^ one day prior to infection. Spleens were harvested on day 18 p.i. PbTII x 10BiT CD4 T cells (1x10^6^) were CTV labeled and adoptively transferred to WT recipients one day prior to infection. Spleens were harvested on day 4 p.i.

### Mixed bone marrow chimeras

For 1:1 chimeras, WT recipient (CD45.1) mice were irradiated with 6.5 and 5.5 Gy, separated by 12 hours. Bone marrow from WT (CD45.1) and *Il10rβ*^-/-^ (CD45.2) were mixed 1:1 and 10^7^ cells were injected i.v. Chimerism was assessed at 5 weeks and mice were infected with *P*. *yoelii* at 6-weeks post reconstitution. For B^WT^ and B^IL10R-/-^ chimeras, WT (CD45.2) mice were irradiated with 6.5 and 5.5 Gy, separated by 12 hours. CD45.1 bone marrow from WT or *Il10rβ*^*-*/-^ and μMT (CD45.1/2) were mixed 1:9 and 10^7^ cells were injected i.v. Chimerism was assessed at 6 weeks and mice were infected with *P*. *yoelii* at 8 weeks. For B cell-specific deletion of specified genes, chimerism for all mice was >80%. All chimeric mice were maintained on oral sulfamethoxazole for 2 weeks after irradiation.

### Confocal microscopy

Spleens were fixed in zinc formalin, dehydrated in sucrose, embedded in tissue freezing media (General Data Healthcare), and cryosectioned at 10um thickness. Mounted sections were permeabilized with 0.4% Triton X-100 in PBST for 15 minutes, blocked with 1% BSA in PBST for 1.5 hours, and stained with either rabbit anti-mouse IL10 primary antibody (Abcam), anti-B220-AF488 (eBioscience), anti-GFP-AF488 (BioLegend), anti-RFP-biotin (Abcam), anti-Thy1.1/PE-Dazzle (BioLegend), anti-CD4-AF594 (BioLegend) or anti-CD4-BV421 (BioLegend) for 24 hours in PBST and counter stained with either streptavidin-PE/Dazzle 594 (BioLegend) or anti-rabbit-IgG-AF594 (BD Biosciences) for 1.5 hours, as specified in the figure legends. Sections were visualized under a Zeiss LSM-710 confocal microscope at indicated magnifications and the images were processed using IMARIS software (Bitplane). For IL-10-expressing cell localization, spheres were created for Thy1.1 signal and manually quantified and scored as either follicular or extrafollicular.

### Single Cell RNA sequencing and computational analyses

Naïve B cells (CD19^+^CD138^-^IgD^+^), plasmablasts (CD19^+^CD138^hi^IgD^neg^), and GC B cells (CD19^+^GL7^+^Fas^+^) from day 10 *P*. *yoelii*-infected mice (n = 3) were sorted (BD FACSAria II). Cells were partitioned into nanoliter-scale Gel Bead-In-EMulsions (GEMs) to achieve single cell resolution for a maximum of 10,000 individual cells per sample. Utilizing the Chromium Single Cell 5’ Library & Gel Bead Kit (10x genomics, 1000006), poly-adenylated mRNA from an individual cell was tagged with a unique 16 base pair 10x barcode and 10 base pair Unique Molecular Identifier. The cDNA amplicon size for the library (~700 bp) was optimized using enzymatic fragmentation and size selection and the concentration of the 10x single cell library was determined (Agilent High Sensitivity DNA Kit (Agilent). The single cell 5’ libraries were sequenced on the Illumina HiSeq-4000 sequencer (The NUSeq Core facility, Northwestern University) at 26 x 98 bp, paired end to target more than 50,000 reads per cell. We adopted Cell Ranger 3.0.2 (https://support.10xgenomics.com/single-cell-gene-expression/software/pipelines/latest/what-is-cell-ranger) for raw sequencing process. All raw short reads were assembled by Cell Ranger and then the assembled reads were mapped to mm10 mouse genome. Using the Cell Ranger output, we performed transcriptome analyses using Seurat 3.0 [54], trajectory and pseudotime analysis using SCANPY-PAGA (Partition-based graph abstraction, version 1.5.0) [55,56]. Seurat 3 was used for cell quality control, data normalization, data scaling, dimension reduction (both linear and non-linear), clustering, differential expression analysis, batch effects correction and data visualization. Unwanted cells were removed according to number of detectable genes (no. of genes < 200 or > 2500 will be removed). Transcriptome data were normalized by a log-transform function with a scaler factor 10,000. We used variable genes in principal component analysis (PCA), and used top 30 principal components (PCs) in non-linear dimension reduction and clustering. All high-quality cells were then clustered by a community detection clustering method implemented in Seurat 3. Differentially expressed genes for each cell cluster were identified Wilcoxon rank-sum test implemented in Seurat 3. Batch effects correction analysis was performed using an “Anchor” method implemented in Seurat 3 to remove the batch effects among different datasets. Developmental trajectory and diffusion pseudotime were built using Scanpy PAGA. “Cluster 1” was set as the root for diffusion pseudotime analysis. Cells and trajectory were visualized on a 2D embedding using ForceAtlas2 (FA) algorithm [57] implemented by SCANPY-PAGA.

### Statistical analysis

Statistical analyses, end point titers, and overall parasite burden, represented as area under the curve for rodent studies were performed using GraphPad Prism 6 software (GraphPad). Specific tests of statistical significance are detailed in the figure legends.

## Supporting information

S1 Fig Lack of parasite control, GC B cell reactions, and humoral immunity in *P*. *yoelii*-infected IL10^-/-^ mice. (**A**) GC-TFH cells were sort-purified from WT and *Il10*^-/-^ mice on day 10 p.i. and mRNA levels of *Il10* were determined using RT-PCR. (**B-F**) WT and *Il10*^-/-^ mice were co-housed for one week prior to infection with 1x10^6^ P. *yoelii* parasitized red blood cells (pRBC). (**A**) Parasitemia (% of infected RBC) in mice surviving through day 18 p.i. (n = 7 *Il10*^-/-^, n = 15 WT). (**B**) Survival of *P*. *yoelii*-infected WT and *Il10*^-/-^ mice. Representative plots and summary data of GC B cells (GL7^+^Fas^+^) (**C**) and class-switched (IgD^neg^IgM^lo^) GC B cells (**D**) in WT and *Il10*^-/-^ mice on day 10 p.i. (**E**) End point titers of parasite-specific IgM, IgG1, and IgG2b on day 18 p.i. Data (means ± S.E.M.) in **B**-**E** are pooled from 3 independent experiments. Data in **A** were analyzed by multiple t-tests correcting for multiple comparisons using the Holm-Sidak method. Data in **B** were analyzed by a Log-rank (Mantel-Cox) test. Data in **C**-**E** were analyzed using unpaired, non-parametric Mann-Whitney tests. * P ≤ 0.05, *** P ≤ 0.001, **** P < 0.0001.
(EPS)

S2 Fig Characterization of IL-10 expressing cells in *P*.*yoelii*-infected mice. (**A-C**) 10Bit (Thy1.1)-IL-10 reporter mice were infected with 10^6^ *P*. *yoelii* pRBC. Gating strategy (A) is shown for two representative mice on day 4 p.i., highlighting variability among IL-10-expressing CD19^+^MHCII^+^ B cells. Summary data showing the relative proportions (**B**) and total numbers (**C**) of splenic Thy1.1^+^ T cells and non-T cells. Data in B,C are pooled from two independent experiments. (**D**) Relative engraftment of WT and *Il10*^-/-^ CD4 T cells in *Tcrα*^-/-^ recipient mice on day 18 p.i. (**E**) WT:*Il10rβ*^-/-^ (1:1) mixed bone marrow chimeric mice (n = 8 mice/group) were generated and infected with 10^6^ *P*. *yoelii* pRBC. Representative plots (left) and summary data (right) of TFH and GC-TFH cells on day 10 p.i. Lines connect WT and *Il10rβ*^-/-^ cells in the same mouse. Data were analyzed using paired, non-parametric Wilcoxon matched-pairs signed rank test and are representative of three experiments.
(EPS)

S3 Fig Localization of IL-10-expressing cells in *P*. *yoelii*-infected mice. (**A**) IL-10-eGFP/Foxp3-RFP double reporter mice were infected with *P*. *yoelii*. On day 4 p.i., spleens were and stained with anti-GFP-AF488 (green), anti-RFP-biotin, anti-CD4-AF647 (white), and B220-BV421 (blue). The sections were then counter-stained with streptavidin-PE/Dazzle (red). Sections were visualized under a Zeiss LSM-710 confocal microscope. Fo, follicle. (**B,C**) Histological examination of IL-10 production on day 4 p.i. in the spleens of 10BiT mice stained with anti-B220-AF488 (green) anti-CD4-AF647 (blue) and anti-Thy1.1-PE/Dazzle (red). (**D**) WT C57BL/6 mice were infected with *P*. *yoelii* and on day 3.5 p.i. and spleens were stained with rabbit anti-mouse IL-10, anti-B220-AF488 (green), anti-CD4-BV421 (white) and counter-stained with anti-rabbit IgG-AF594 (red). Images in A and D are representative of 5 sections examined from double reporter (n = 3) and WT (n = 2) spleens and were processed using IMARIS software. Images in B and C are representative of 4 independent tiled scans acquired from n = 4 10Bit spleens. Scale bars in primary and inset images in A and D represent 50 μm and 20 μm, respectively. Scale bars in B and C represent 200 μm and 50 μm, respectively. (**E**) Summary of absolute number of IL-10/eGFP+ CD4 T cells that localized outside and within B cell follicles. Data in C were analyzed using an unpaired, non-parametric Mann-Whitney test. *** P < 0.0001.
(TIF)

S4 Fig Phenotype of B cells activated in the absence of IL-10. (**A**-**C**) *P*. *yoelii*-infected mice were treated with anti-IL-10R mAb from days 2–4 p.i. Representative histograms (**A**), total numbers (**B**), and geometric mean fluorescence intensity of Ki67 expression (**C**) in IgD^neg^CD62L^lo^ activated B cells on day 4 p.i. (**D**) *March1* mRNA expression in sort-purified activated follicular (CD19^+^B220^+^CD23^+^CD21/35^neg^IgD^neg^CD62L^lo^) B cells recovered from rIgG- and anti-IL-10R-treated mice in day 6 p.i. as measured by qPCR. Data show fold change as normalized to rIgG-treated controls. Data in A-C are from n = 3 mice/group and representative of two independent experiments. (**E**,**F**) WT (n = 4) and *Il10*^-/-^ (n = 4) mice were infected with *P*. *yoelii*. Spleens were harvested on day 9 p.i. (**E**) Representative plots and summary data showing the frequency and total number of IgD^neg^CD62L^lo^ activated B cells. (**F**) Summary data showing frequency and total numbers of activated B cells expressing SLAM (left panels), MHCII (middle panels) and CD83 (right panels) on day 9 p.i. Data (means ± S.E.M.) in E-F were analyzed using unpaired, non-parametric Mann-Whitney tests. * *P* ≤ 0.05, ** *P* ≤ 0.01.
(EPS)
