# Supplementary figures and images for: Extrafollicular CD4 T cell-derived IL-10 functions rapidly and transiently to support anti-Plasmodium humoral immunity
Source: PLoS Pathog. 2021 Feb 2;17(2):e1009288. doi: 10.1371/journal.ppat.1009288 (PMC7880450; doi:10.1371/journal.ppat.1009288)

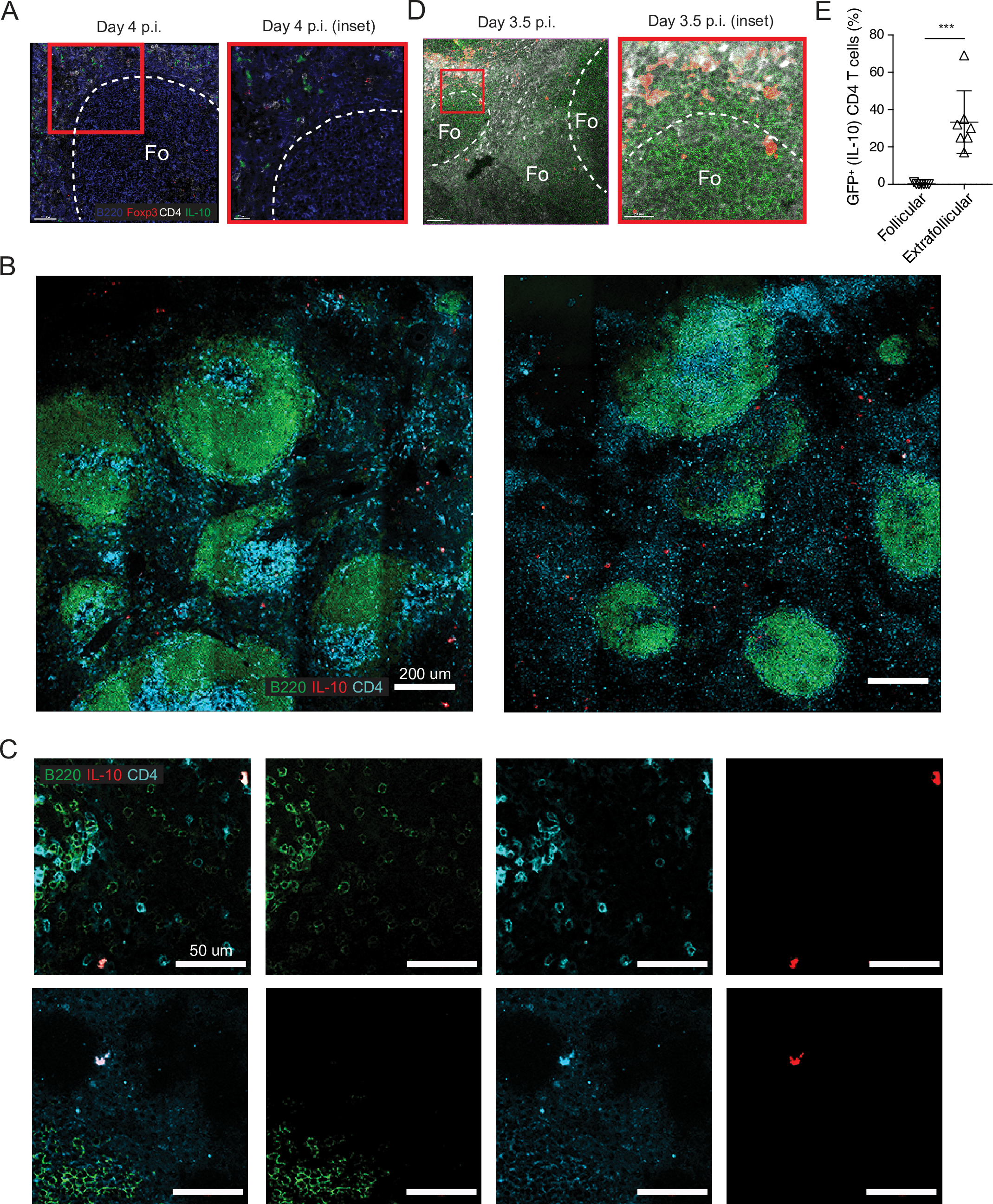

Supplement: S3 Fig — (A) IL-10-eGFP/Foxp3-RFP double reporter mice were infected with P. yoelii. On day 4 p.i., spleens were and stained with anti-GFP-AF488 (green), anti-RFP-biotin, anti-CD4-AF647 (white), and B220-BV421 (blue). The sections were then counter-stained with streptavidin-PE/Dazzle (red). Sections were visualized under a Zeiss LSM-710 confocal microscope. Fo, follicle. (B,C) Histological examination of IL-10 production on day 4 p.i. in the spleens of 10BiT mice stained with anti-B220-AF488 (green) anti-CD4-AF647 (blue) and anti-Thy1.1-PE/Dazzle (red). (D) WT C57BL/6 mice were infected with P. yoelii and on day 3.5 p.i. and spleens were stained with rabbit anti-mouse IL-10, anti-B220-AF488 (green), anti-CD4-BV421 (white) and counter-stained with anti-rabbit IgG-AF594 (red). Images in A and D are representative of 5 sections examined from double reporter (n = 3) and WT (n = 2) spleens and were processed using IMARIS software. Images in B and C are representative of 4 independent tiled scans acquired from n = 4 10Bit spleens. Scale bars in primary and inset images in A and D represent 50 μm and 20 μm, respectively. Scale bars in B and C represent 200 μm and 50 μm, respectively. (E) Summary of absolute number of IL-10/eGFP+ CD4 T cells that localized outside and within B cell follicles. Data in C were analyzed using an unpaired, non-parametric Mann-Whitney test. *** P < 0.0001. (TIF) [file ppat.1009288.s003.tif]
